# Efficacy of the use of video games on mood, anxiety and depression in stroke patients: preliminary findings of a randomised controlled trial

**DOI:** 10.1007/s00415-023-12043-z

**Published:** 2024-01-10

**Authors:** Patricia Blázquez-González, Rubén Mirón-González, Alejandro Lendínez-Mesa, Raquel Luengo-González, Noelia Mancebo-Salas, María Teresa Camacho-Arroyo, Alfonso Muriel-García, María Montserrat García-Sastre

**Affiliations:** 1 Department of Nursing, Red Cross University, Madrid, Spain; 2 Biomechanics and Bioengineering Applied to Health, Doctoral School, University of Alcalá, Madrid, Spain; 3 Group for Research in Community Care and Social Determinants of Health, University of Alcalá, Madrid, Spain; 4 Nursing and Physiotherapy Department, University of Alcalá, Madrid, Spain; 5 Departamento de EnfermeríaServicio de Neurología, Hospital Universitario 12 de Octubre, Madrid, Spain; 6 Group for Research in Nursing Care, Gregorio Marañón Health Research Institute (IiSGM), Madrid, Spain; 7 General Directorate of Social Services With the Ministry of Family, Youth and Social Policy of the, Community of Madrid, Madrid, Spain; 8 Primary Care Center Sierra de Guadarrama SERMAS, Madrid, Spain; 9 Clinical Biostatistics Unit, Hospital Universitario Ramón y Cajal (IRYCIS), CIBER Epidemiología y Salud Pública (CIBERESP), Madrid, Spain

**Keywords:** Anxiety, Depression, Virtual reality, Stroke

## Abstract

**Background:**

In the different published studies, there is no consensus on the efficacy of virtual reality as an adjuvant treatment of mood states.

**Aim:**

The aim of this study is to evaluate the impact of no immersive virtual reality with the Nintendo Switch device in rehabilitation treatment on mood, anxiety and depression in stroke patients admitted to neurorehabilitation units.

**Methods:**

Fifty-eight patients admitted to neurorehabilitation units underwent a 1:3 multicentre randomised clinical trial. The intervention group consisted of 17 patients and the control group of 41 patients. The intervention group performed 6 virtual reality sessions together with the conventional treatment, and the control group performed only the conventional rehabilitation sessions. Primary and secondary clinical outcomes were measured before and six weeks after the intervention.

**Results:**

Comparing the intervention group and control group, the anxiety levels of the intervention group decreased compared to the results observed in the control group (*p* = 0.01), as did the dependence of the intervention group (0.015). On the other hand, the results obtained after the intervention by the control group for anxiety (0.479) and depression (0.292) were not statistically significant.

**Conclusion:**

Rehabilitation VR used as an adjuvant treatment to conventional treatment has a beneficial impact on the neurological status and state of anxiety of stroke patients admitted to neurorehabilitation units.

**Trial registration:**

Registered in the https://clinicaltrials.gov/ repository (NTC NCT05143385). Protocol registration date 7 October 2021, retrospectively registered.

## Introduction

Stroke is the leading cause of disability and the second leading cause of death worldwide. Amongst stroke patients, 30% require daily assistance, so early care by multidisciplinary teams can reduce dependency in these patients [1]. It is estimated that 77% of survivors have chronic sensorimotor deficits that affect functional independence [2].

High morbidity and mortality rates associated with stroke have led to the creation of specialised units for its treatment [3].

People who have suffered a stroke can experience a variety of psychological consequences, anxiety and depression that can compromise functional status [4] and the rehabilitation process in the long term. After a stroke, anxiety and depression are common symptoms [5, 6].

The prevalence of anxiety is 29.3% during the first year, and depression rates reach 33% [5, 6]. Both diseases are associated with higher levels of disability and dependency [7]. Depression after stroke is considered the main predictor of poor functional outcome after stroke and has a strong interference with rehabilitation. Post-stroke patients with gait disturbance and with dependence for the development of activities of daily living (ADLs) have a greater state of depression than autonomous patients [7].

Patients with anxiety have feelings of boredom, frustration, worry, despair and guilt that make the stroke patient feel uncomfortable. A low level of anxiety is described as a problem or worry, and a high level of anxiety appears as panic or fear, manifesting itself physically as stress [6].

Depression after stroke is characterised by pessimism, despair, sleep disorders and inferiority. These characteristics influence the rehabilitation period. Current research on the functioning of the emotional state is focussed on imaging techniques since they provide information on the brain network, structural changes, functional connexions and excitability of all brain areas. Studies have shown that emotional disturbances are caused by severe trauma and nerve injury due to stroke and that the severity of the stroke may or may not be a key factor in the development of mood disturbance [8].

Anxiety is difficult to diagnose in stroke patients. This is due to the difficulty in differentiating between normal worries and the appearance of pathological anxiety disorders or significant levels of anxiety. In stroke patients, advanced age and limited verbal communication ability make it difficult to diagnose anxiety; therefore, patients do not receive treatment [9].

In recent years, virtual reality (VR) has demonstrated its usefulness in mobility, balance and gait as an adjunctive treatment to conventional treatment [10]. VR plays an important role in the treatment of anxiety disorders in the neurological patient. It is able to improve levels of well-being, coping strategies and social relationships [11]. In non-immersive virtual VR, the most widely used game consoles are the Wii, Xbox, Jintronix Rehabilitation System^™^ and PlayStation [10, 12].

New VR systems allow stimulus control within a dynamic, multisensory environment, creating a safe and motivating exercise condition that can prevent the injury and exhaustion that could occur when performing in a real environment. Another advantage is the fact that regular exercise has a positive impact on depression, anxiety and other mental disorders [13].

Stroke patients are often isolated in their daily life from their family and society. The use of VR in rehabilitation allows the patient to obtain immediate feedback on task performance and visual and audible stimulation. It also often arouses the patient’s interest. VR generates motivation in treatment participation, and patients have fun whilst actively performing tasks. It can provide stability in ADLs in which they were previously limited, thus implementing autonomy for the stroke patient [8, 11, 13].

VR is considered a cost-effective tool for the treatment of psychological disorders and specifically for the treatment of anxiety. Studies reviewed claim that VR has positive effects on the affective state of patients and may become a more cost-effective treatment than traditional anxiety treatments. There is no evidence to support the use of VR as a stand-alone treatment for mood disorder, anxiety and depression [11, 13, 14].

In addition to the improvement of functional status, VR used in the treatment of depression in stroke patients improves the state of depression and interpersonal relationships when used in a group setting [13].

Nurses, in addition to their performance in the functional rehabilitation of stroke patients, have a fundamental role in the detection of neuropsychiatric problems in patients.

Their continuous working day of 24 h a day or 7 days a week, favours them to be the first professionals to detect signs or symptoms associated with depression [15]. In addition, nurses have a fundamental role with their intervention in improving depression and anxiety in stroke patients [16].

Because of this care relationship, nurses must be involved in their re-education and monetization [15]. In addition, nurses have a key role with their intervention in improving depression and anxiety in stroke patients [16].

The aim of this research study is to investigate the efficacy of VR used as a complementary treatment on mood, anxiety and depression in patients admitted to neurorehabilitation units by comparing these patients to stroke patients who only received conventional rehabilitation.

The secondary objective is to investigate whether VR intervention influences the improvement of brain damage and whether it has an impact on the mobility of patients who have suffered a stroke by comparing these patients to those who have only undergone conventional rehabilitation.

## Methodology

### Study design

This study was designed as a multicentre, randomised, controlled trial conducted in two rehabilitation hospitals in the city of Madrid (Spain): Fundación Instituto San José (FISJ) and the Centro Estatal de Atención al Daño Cerebral (CEADAC). The FISJ is a hospital specialising in neurological and traumatological rehabilitation. Reference in Palliative Care. The CEADAC is a public social and health centre for the comprehensive and interdisciplinary rehabilitation of users with acquired and non-progressive brain damage.

Each hospital was responsible for the recruitment, assessment and therapy of all participants. Forty-five patients were recruited in the FISJ and 13 in the CEADAC, after they had been hospitalised for a minimum of 15 days to ensure that they were aware of the environment and the operation of the unit in each hospital.

**Fig. 1:**
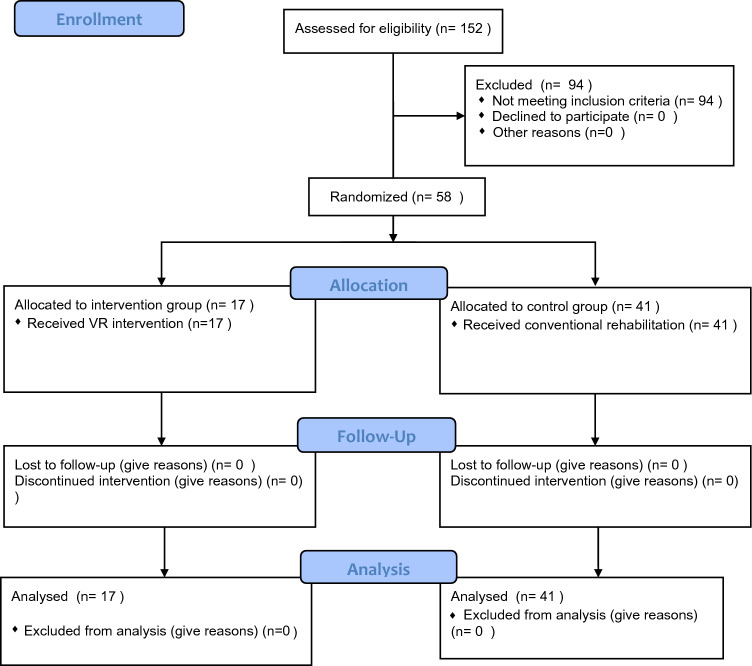
Flow diagram of the study procedure

More details regarding the study methodology can be found in the study flow chart in Fig. 1.

The Clinical Research Ethics Committee of the Hospital Clínico San Carlos of Madrid approved the study protocol on July 1, 2021.

The CONSORT 2010 statement [17] was used to conduct and report the results of this trial.

### Participants

The inclusion criteria for participation in the study were that the patients 1) had suffered a stroke, 2) were of legal age, 3) had a command of oral and written Spanish, which was measured [18–21], 4) were able to maintain cognitive functions of expression and comprehension contrasted through the MAST scale and the Pfeiffer scale [18–22], 5) had not started (15 days) neurorehabilitation treatment and 6) had mobility in at least one upper limb and were able to maintain a sitting position. The exclusion criteria were that patients 1) were not admitted to the neurorehabilitation programme, 2) had a history of a psychiatric disorder and 3) had loss of visual acuity.

The patients were invited to a meeting in which researchers provided them with comprehensive information about the objectives of the study and its development. Those who decided to participate in the study were randomised. Patients were provided with a patient information sheet outlining the parameters and recruitment information already explained at the initial meeting. In addition, they were provided with an email from the principal investigator, to whom they could ask any questions.

### Randomisation and masking

Allocation was performed in a simple randomised fashion, in a 1:3 ratio like a reviewed study [7], through the Excel 2019 tool. As this was an interventional clinical trial, both the patients and the professionals knew about the control group (CG) and the intervention group (IG), and masking was not contemplated. Blinded third-party assessment was performed in the data analysis and allocation concealment.

### Procedures

Patients in the CG and IG underwent conventional rehabilitation, which varied depending on the centre. In the FISJ, it consisted of: 400 min per week of individualised physiotherapy, 100 min per week of occupational therapy sessions, 120 min per week of computer therapy, 90 min per week of sessions with the neuropsychologist and speech therapy sessions, depending on the patient, which ranged from 90 to 120 min per week. In the CEADAC, physical therapy sessions lasted 600 min per week, with physical therapy exercises and individualised exercises and therapies performed together with other patients within that schedule; occupational therapy sessions lasted 120 min per week, with some therapies performed together with other patients; neuropsychology sessions were 30–60 min depending on the patient and speech therapy sessions were 30–60 min depending on the individualised treatment of the patient.

Conventional rehabilitation began after assessment by the rehabilitation physician on the day of admission. The adjuvant treatment with VR performed by the IG began for all participants 15 days after admission and was prolonged in both centres to 6 sessions of 20 min once a week. This intervention was performed in both centres in a private room. Intervention description was based on the template for intervention description and replication (TIDieR) checklist and guide [23] and *ClinicalTrials.gov* recommendations (Table 1).

**Table 1:** Description and replication study intervention description-based *ClinicalTrials.gov*

Item		IG ^a^	CG ^b^
1	Brief name	VR^c^ + CR^d^	CR^d^
2	Why	Both interventions were compared in stroke patients to demonstrate that virtual reality-based technology improves anxiety and depression in hospitalised patients. If so, it could be implemented as an adjuvant therapy in specialised neurorehabilitation units	
3	Pre-assignment Details	A briefing session was held with both groups to explain what the study consisted of and what the GI and CG rehabilitation would consist of. In addition, participants who freely agreed to participate in the study signed the informed consent and patient information sheet The e-mail address of the principal investigator was provided We began by reading the clinical history of the patients who agreed to participate and performed the inclusion and exclusion criteria to obtain the sample The information was obtained from the clinical histories and the tests indicated in Table 2 were performed	
4	Arm/Group Information	The participants were randomised using the computer programme	
5	Arm/Group Title Arm/Group Description	During the first session, a 10 min explanation of the use of the game console was given Patients in this group were moved once a week to a VR room where the Nintendo Switch device was located In addition, this group underwent individualised conventional rehabilitation consisting of functional rehabilitation, occupational therapy, computer therapy, neuropsychologist and speech therapist	Performed: functional rehabilitation, occupational therapy, computer therapy, neuropsychologist and speech therapist
6	Period(s)	The VR intervention lasted 20 min, once a week, for 6 weeks Conventional therapy is performed daily with an individualised duration for each patient	Conventional therapy is performed daily with an individualised duration for each patient
7	Study participants	Beginning: 17 participants, 4 participants belonging to CEADAC and 13 participants belonging to FISJ Completion: all participants completed the study	Beginning: participants, 13 participants belonging to CEADAC and 32 participants belonging to FISJ Complete: all participants completed the study
8	Finals test	The tests indicated in Table 2 were performed	

^a^Intervention group

^b^Control group

^c^Virtual reality

^d^Conventional rehabilitation

The VR sessions were led by two nurse researchers with expertise in neurorehabilitation who have more than 15 years of experience in the care of neurological patients and who accompanied and supported the patients throughout the intervention. They received a training session, given by the principal investigator, for the handling of the VR device following the protocol designed for the implementation of the intervention in this trial. The VR intervention was performed with the Nintendo Switch device and the video game Mario Party, which consists of different competitive and fun mini-games, where the patient has to use only one upper limb (MS) and perform flexion–extension movements of the elbow and pronation-supination of the forearm.

### Outcomes

Demographic data were obtained from the medical records. The scales used were: the Pfeiffer scale [22], the Volume–Viscosity Swallow Test (V–VST) [24] and the Mississippi Aphasia screening test [18–20]. The data for the secondary objective were obtained through the Barthel Index [25, 26] and the Canadian scale [27, 28] measured before and after the intervention, and the main objective was obtained through the Hospital Anxiety and Depression Scale (HADS) [29, 30] and after the intervention (Table 2).

**Table 2:** Outcome measures and measurement sessions

Assessment	Abbreviation	Outcome	Measurement sessions	
Primary outcome			B0^a^	PI^b^
Hospital Anxiety and Depression Scale	HADS	Anxiety Depression	x	x
			x	x
Secondary outcome				
Barthel Index	BI	Independence in ADL^c^	x	x
Canadian scale	C	Neurological assessment	x	x
Outcome for descriptive outcomes				
Pfeiffer^b^	P	Detect cognitive impairment	x	
Volume–Viscosity Swallow Test	(V–VST)	Identify oropharyngeal dysphagia	x	
Mississippi Aphasia screening test	MAST	Aphasia detection	x	

^a^Baseline

^b^Post-intervention

^c^Activities of daily living

### Data analysis

Through the estimation formula of proportions, taking a confidence level of 95%, a precision of 3%, a margin of error of 0.05 and a loss of 10% of data of patients to be excluded from the study, considering the data provided by the admission service of the FISJ and CEADAC, the required sample size was 45 in the FISJ and 13 in the CEADAC.

### Analysis of outcomes

The participants’ data were entered into the database by researchers outside the intervention, guaranteeing blinding in the handling of the data by separating the values of the measurements from the general information of the participants.

Descriptive statistics of demographic and clinical characteristics were used for baseline results of the outcome measures in both groups. Descriptive statistics, including means and standard deviation and relative frequencies for the study participants are presented.

To determine the statistical test for use in data analysis, we first evaluated the normal distribution of the data using the Kolmogorov–Smirnov test [31]. The chi-square test was used to compare the initial baseline data regarding the variables, type of stroke, main caregivers, antidepressant use, neuroleptics and clinical information on whether the patients have dyslipidaemia, are hypertensive or have metabolic diseases, Canadian scale and the volume–viscosity test. For the rest of the variables, the Student’s *t* test was used when the variables had a normal distribution and for the non-parametric variables, the Wilcoxon-Mann-Whitney test was used for unrelated samples and the Wilcoxon test was used for related samples.

The analysis was conducted in three parts for functional status and mood state. First, scores on each outcome measure at each time point (before the start of the intervention and after the intervention) were compared between the CG and the IG using Wilcoxon-Mann-Whitney for independent samples. Second, for each treatment group, the significance of change between pre-test and post-test on each outcome measure was analysed using Wilcoxon. Third, correlation tests were used to test the correlation between baseline and endpoints of the Barthel Index scale with the HADS scale and the Canadian scale with the HADS scale. For this analysis, the Barthel Index variable and the Canadian Scale were considered as the independent variable and the anxiety and depression variable as the dependent variable.

Results were considered statistically significant at *p* < 0.05. Analyses will be performed using Statistical Package for Social Sciences version 26 8 IBM, 290 M (Armonk, New York, USA). Finally, the statisticians performing the analyses were blinded to participant assignment.

## Results

Between October 2021 and January 2022, a total of 58 participants were recruited as planned at the two centres involved in the study. A total of 17 patients were randomly assigned to the VR group, of whom 4 were from the CEADAC (4 men) and 13 from the FISJ (11 men and 2 women). The CG consisted of 41 patients, 9 from the CEADAC (3 men and 6 women) and 32 from the FISJ (19 men and 13 women). All the participants completed the study (Fig. 1), but the sample could not be completed in the IG due to the COVID-19 pandemic, following the recommendations of the hospital contingency plan.

Of the patients who formed the IG, only two patients reported sporadically playing video games, with the rest of the patients reporting no previous interest in the use of VR.

The sample consisted mostly of patients who had suffered an ischaemic stroke (64%) and, to a lesser extent, of patients who had suffered a haemorrhagic stroke (35%). There was no differentiation in stroke subtypes as data were not specifically collected in the medical records of all patients in the study. Table 3 shows the demographic and baseline clinical characteristics of the IG and CG of the two centres. Baseline characteristics of the different groups were similar, except sex and taking antidepressants, for time since stroke was higher in the CEADAC (77.38 $\pm 45.43$) compared to FISJ (68.11 $\pm 34.66$). All changes in scores of the primary and secondary outcomes are provided in Tables 4 and 5.

**Table 3:** Demographic and clinical characteristics. Values are presented: in means ± SD and relative frequencies

	IG^a^ (*n* = 17)	CG^b^ (*n* = 41)
Age	54.23(6,53)	54.29(7,19)
Sex	0,88	0,53
Type of Stroke Ischemic Haemorrhagic	0.64 0.35	0.48 0.51
Main Caregivers Spouse Parents Siblings Children No caregiver needed	0.58 0.17 0 0.17 0.58	0.51 0.73 0.12 0.29 0
Volume–Viscosity Swallow Test Solid Liquids Nectar Pudding Without dysphagia	0.58 0 0.58 0 0.88	0 0.24 0.21 0.24 0.73 0
Time since Stroke	74,47($\pm \mathrm{36,21})$	67,54($\pm 38,04)$
Physical therapy time	805,29($\pm$ 154,693)	893,41($\pm$ 164,873)
Antidepressants	0.35	0.97
Neuroleptic	0.11	0.31
Dyslipidemia	0.76	0.63
Hypertensive	0.76	0.76
Metabolic diseases	0.35	0.36

^a^Intervention group

^b^Control group

**Table 4:** Primary outcomes

	IG	IG	p-value^*^	CG	CG	p-value^*^	P-value^**^ Baseline IG vs. CG	P-value^**^ Post-intervention IG vs. CG
	Baseline	6 weeks		Baseline	6 weeks			
HADS Anxiety	6.1($\pm$ 4)	2.5 ($\pm$ 2.5)	< 0.01	6.7($\pm$ 4.1)	7.4($\pm$ 4.5)	0.479	0.751	< 0.001
HADS Depression	7.2($\pm$ 4.7)	5.71 ($\pm$ 3.8)	0.115	7.8($\pm$ 4)	8.9($\pm$ 3.9)	0.292	0.526	0.001

*IG* Intervention Group, *CG* Control Group, *HADS* Hospital Anxiety Depression Scale

*p-value: Wilcoxon, **p-value: Wilcoxon-Mann-Whitney

**Table 5:** Details of secondary outcomes

	IG Baseline	IG 6 weeks	p-value	CG Baseline	CG 6 weeks	p-value	P-value Baseline IG vs. CG	P-value Post-intervention IG vs. CG
Canadian scale Level Moderate	70.5 29.4	100 0	0.015^*^	60.9 39.0	85.3 14.6	0.013^*^	0.488^*^	0.096^*^
Barthel	57 ($\pm$ 28.4)	75 ($\pm$ 29.5)	0.013^**^	54 ($\pm$ 27.9)	59.2 ($\pm$ 32.3)	0.487^**^	0.681^***^	0.083^***^

*p-value: obtained with the Chi-square test, **p-value: Wilconxon, ***p-value: Wilconxon-Mann-Whitney

## Primary outcome evaluation

### Mood state

#### HADS anxiety

Pre-study values for anxiety levels measured with the HADS anxiety scale reported similar values in the CG and IG (6.1($\pm$ 4) vs. 6.7($\pm$ 4.1), *p* = 0.751). There were no statistically significant differences in independence (*p* = 0.751) between the IG and CG (Table 4). The IG participants obtained lower anxiety values after the intervention (6.1 $\pm$ 4 versus 2.5($\pm$ 2.5), *p* =  < 0.01) than the CG (6.7 ($\pm$ 4.1), versus 7.4 ($\pm$ 4.5), *p* = 0.479). The IG, moreover, obtained statistically significant differences after the intervention (*p* =  < 0.01) (Table 4).

#### HADS depression

Pre-study values for depression levels measured with the HADS depression scale reported similar values in the CG and IG before intervention (7.2($\pm$ 4.7) versus 7.8($\pm$ 4) (*p* = 0.526). There were no statistically significant differences in independence (*p* = 0.526) between the IG and CG groups (Table 4). IG participants obtained lower depression values after the intervention (7.2($\pm$ 4.7) versus 5.7($\pm$ 3.8) *p* = 0.115) than the CG (7.8($\pm$ 4) versus 8.9 $\pm$ 3.9), *p* = 0.292). There were no statistically significant differences (*p* = 0.292) (Table 4).

There were also no statistically significant differences in independence between baseline IG and CG, but there were statistically significant differences after the intervention between the IG and the CG (0.001).

## Secondary outcome evaluation

### Canadian scale

On the Canadian scale, there were no significant differences in the impact of the stroke (*p* = 0.488) between both groups. On the other hand, the V–VST did not show statistically significant differences between both groups either (Table 5).

### Functional status

At baseline, the IG reported higher Barthel scale scores than the CG (57($\pm$ 28.4) versus 54($\pm$ 27.9), *p* = 0.681). There was no statistically significant difference in independence (*p* = 0,681) between the intervention and CG at baseline (Table 5).

IG participants reported a decrease in dependence and statistically significant differences after the intervention (57($\pm$ 28.4) versus 75($\pm$ 29.5), *p* = 0.013) (Table 5). CG participants did not improve independence and showed no statistically significant differences (54($\pm$ 27.9) versus 59.2($\pm$ 32.3), *p* = 0.487). There were also no statistically significant differences regarding independence between all participants before and after the intervention (0.487).

If we compare the results obtained before and after the intervention between the IG (*p* = 0.681) and the CG (*p* = 0.083), no statistically significant results were obtained.

## Discussion

As we hypothesised, the IG has significantly improved their anxiety scores, with no statistically significant results for the depression variable, after the 6-week VR intervention.

The mental state of stroke patients is associated with higher levels of dependency and disability [32, 33]. In this research, data obtained 15 days after admission showed that 58.62% of patients had anxiety and 60.34% had depression. These data no coincide with the systematic review and meta-analysis that report lower levels of anxiety [6] and depression in stroke [34]. Furthermore, it is essential to take into account the emotional regulation of each individual, which influences their experience and expression of emotions, playing an important role in the achievement of goals and influencing psychosocial well-being and the improvement of mental health [35]. These data may be explained by the underestimation of anxiety and depression in patients with these characteristics [6].

These data can be explained if we frame the study within the time of the COVID-19 pandemic in which it was carried out since different studies claim higher anxiety and depression scores in stroke patients. [36, 37]. These data may explain the increased use of antidepressants found in this study (79.31%) compared to previous studies where they only represent 50% of diagnosed patients [38].

According to the hypothesis, our study confirms that post-stroke rehabilitation supported by adjuvant VR treatment has positive impacts on mood, decreasing anxiety levels after 6 weeks of VR intervention. These results coincide with different published studies [7, 39]. On the other hand, it does not seem to be effective in the treatment of depression, coinciding with another study framed in the same context [39] and other studies that show benefits as an adjuvant treatment [40–42]. These results may be due to the intervention not being conducted daily, as the total intervention time was shorter than in other studies [14, 43]. None of the studies similar to our study report on patients' prior knowledge of VR or its use before stroke [7, 14, 40, 43, 44].

If we take into account the sociodemographic variable age, we found studies with mean age higher than that of our study in the intervention group (77.5) and in the control group (63) with the same results regarding the effectiveness of the use of VR as a complementary tool in the treatment of anxiety [7, 45]. Other studies with statistically significant results regarding the effectiveness of VR as a complementary treatment in the treatment of depression in stroke patients that were formed with older patients [40, 42] obtained statistically significant results as in studies where the ages of the patients were younger and more similar to those of the patients who took part in this study [14, 44]. The variable age does not seem to have an influence on the results of the studies reviewed for the effectiveness of VR as adjuvant treatment for anxiety and depression in stroke patients admitted to neurorehabilitation units. The age variable seems to have no influence on the results obtained for the treatment of anxiety and depression in stroke patients admitted to neurorehabilitation units.

Physiotherapy interventions help improve the performance and capacity of the patient, improving neurological and functional involvement [46]. In our study, both the IG and the CG improve neurologically, but the functional improvement was statistically significant for the IG [47, 48]. This may be due to the fact that the period was 6 weeks, and in this type of patient results are not observed in such a short period [49]. Other studies were conducted over a longer duration [50].

This study has several limitations that must be considered: a) lack of long-term follow-up evaluation, b) limited geographic region, c) interventions were infrequent, only once a week, d) the accompanying and support figure of the nurse could have interfered with the results obtained after the intervention. Therefore, the findings should be interpreted with caution, and more repeated measures with longer follow-ups are recommended. This study also has strengths, including its randomised design, the first proposed nurse-led VR training in a neurological care ward, and the 100% study completion rate by participants. Therefore, we recommend that VR be considered as an adjunctive treatment to conventional rehabilitation or for the treatment of anxiety.

As future lines of research, it would be interesting to evaluate the impact of video game use on mood, anxiety and depression in patients with lacunar versus non-lacunar stroke because the pathophysiology, prognosis and clinical characteristics of lacunar stroke are different from other acute cerebrovascular diseases and is one of the most frequent subtypes of ischaemic strokes [51].

## Conclusion

Within the limitations of this preliminary study, VR used as an adjuvant treatment to conventional treatment has a beneficial impact on the functional status and anxiety state of patients admitted to neurorehabilitation centres. The improvement of anxiety and depression levels in patients who have suffered a stroke influences the results of the neurorehabilitation progress and reduces the length of hospital stay and therefore, the cost of hospitalisation.

The Nintendo Switch VR system has been shown to be effective for treating anxiety. This system is a small device that is easy to transport and cheap when compared to other devices on the market.

It should also be taken into account that the accompanying and support figure of the nurse could have interfered with the results obtained after the intervention.

The nursing team has an essential role in the detection of anxiety and depression in hospitalised patients. Early detection of mood disorders helps with treatment of these disorders that hinder the rehabilitation process.

## Data Availability

Data will be made available upon reasonable request to the corresponding author.
